# Key Factors Driving Portuguese Individuals to Use Food Supplements—Findings from a Cross-Sectional Study

**DOI:** 10.3390/foods14050884

**Published:** 2025-03-05

**Authors:** Maria João Campos, Agnieszka Garbacz, Natalia Czlapka-Klapinska, Magdalena Czlapka-Matyasik, Angelina Pena

**Affiliations:** 1 LAQV, REQUIMTE, Laboratory of Bromatology, Pharmacognosy and Analytical Sciences, Faculty of Pharmacy, University of Coimbra, 3000-548 Coimbra, Portugal; mcampos@ff.uc.pt; 2 Department of Human Nutrition and Dietetics, Poznan University of Life Sciences, 60-624 Poznan, Poland

**Keywords:** food supplements, cross-sectional study, consumption, dietary patterns, socioeconomic status, Portugal

## Abstract

Data on food supplement (FS) consumption profiles are scarce. This study aims to characterise FS consumption among Portuguese adults and identify personal, social, professional, and health-related factors influencing FS use. A cross-sectional study was conducted using an online questionnaire between July and September 2023. The participants were categorised into healthcare professionals (supplement users and non-users, i.e., HPS and HPnS) and non-healthcare professionals (supplement users and non-users, i.e., nHPS and nHPnS). Group distributions were compared using the χ^2^ test. FS use is very prevalent in Portugal. Significant differences emerged between HPs and nHPs regarding factors associated with FS use. Socioeconomic and professional characteristics, nutritional knowledge, and healthy lifestyles (e.g., eating habits) were all linked to FS consumption. Despite the differences between the groups, across groups, higher education levels, higher nutritional knowledge, and healthier lifestyle habits, such as engaging in sports and healthy food habits, translate into a higher consumption of FSs. The frequent use of FSs in Portuguese adults is associated with higher education, nutritional knowledge, and healthy lifestyles. HPs have specific attitudes through FS use. These findings indicate the need for further research to explore the various types of FSs being utilised and the underlying motivations behind their usage. HPs’ access to FS scientific information and providing practical guidance to promote responsible and informed FS use within the population is crucial.

## 1. Introduction

Food supplements are significant in public health and nutrition, particularly in addressing nutritional deficiencies and supporting overall well-being. Their use is rising worldwide, and many scientific and regulatory challenges are global.

The European Food Supplements market was valued at USD 14.95 billion in 2019 and is projected to reach USD 53.53 billion by 2032 [1]. FS use is relevant and prevalent in European Union (EU) countries and is growing [2,3]. In 2022, Ipsos, a consulting company, conducted a consumer survey on FSs in the EU, concluding that almost nine in ten (88%) respondents had used an FS at some point in their lives [2]. In Portugal, market studies concluded that the consumption of FSs increased from 12.6% in 2011 to 26.8% in 2023, which is aligned with the Portuguese National Survey data [4,5]. Furthermore, the market for FSs in community pharmacies is growing in Portugal. In 2023, FSs represented a market share of 5.6%, valued at around EUR 256.7 million compared to EUR 230.6 million in 2022 and EUR 192.5 million in 2021 [6,7,8]. There is insufficient information concerning factors driving the Portuguese population to consume FS. Are they health reasons, knowledge, or perhaps advertising? What is published shows the high use of these products. However, our knowledge is mainly concerned with studies on special populations (vegans, athletes) and is not specified for the general population. The factors determining the decision to reach for an FS in the general Portuguese population are unknown.

Some FSs are expected to be important in advancing predictive and proactive health, improving overall health and helping to manage some health conditions [9]. Meanwhile, according to the literature, they are used by the population and recommended by health professionals as health products to prevent or treat clinical conditions, even though, legally, FSs should only be used to complement a healthy diet. They cannot, therefore, have therapeutic indications for the prevention and treatment of diseases but can only claim benefits supported by approved health claims [10,11]. It should be underlined that FSs, according to the literature, are used not only because of their benefits but also because of their easy access, the concern with healthy ageing, and the appealing marketing applied by the companies that distribute these products [12]. Nevertheless, their potential for interaction with medicines worries the scientific community [5,13,14,15].

Additionally, vulnerabilities of the marketing authorisation and the absence of the need for special assistance in their sale can lead to the misuse of these products [3]. These fears associated with FS use by consumers and prescribers highlight the importance of knowing the factors that make a person a potential consumer of these products. There is already some published information. Some studies have shown that using FSs for general health increases with age [2,5]. Data from a study performed in 2015 disclosed that the use of FSs by 26.6% of the Portuguese population was higher in females, adults, and elderly individuals [5]. Similarly, studies in 2018 in Italy showed that the prevalence of “regular” adult consumers was 22.7% of the population [16]. Recent studies performed in 2023 and 2024 analysed the factors that determine the consumption of FSs [17,18,19,20]. The authors considered the athlete population of Portuguese gymgoers and proved the higher prevalence of FS consumption among them [20]. Also, studies related to the COVID-19 pandemic and elderly subjects showed that males with higher energy expenditure in moderate and intense activities and a lower perception of fear of COVID-19 tended to use FSs [19]. A COVID-19 study on medical university students concluded that FSs are commonly used in that population [21]. Information on the prevalence of FS consumption is scarce in the general population [18].

Considering the above situation, no independent surveys have analysed FS users’ complex factors and dietary-related behaviours. Concurrent knowledge of what drives the motives to use FSs is also unknown. It is essential to find the key factors driving Portuguese adults to choose FSs and give data to health associations and authorities to prepare recommendations and guidelines for healthcare professionals and raise awareness among consumers of the risk of FS misapplication due to overuse or FS–drug interactions [12].

There is insufficient research on FS consumption among the general population and significant social and professional groups, including healthcare professionals. Our research aimed to determine the key factors in choosing an FS in a Portuguese adult population, distinguishing between the general population and healthcare professionals.

## 2. Study Sample and Methods

### 2.1. Aims and Research Question

This study aims to characterise FS consumption in Portuguese adults and identify influencing factors (personal, social, professional, and health-related). Furthermore, the researchers also aim to compare FS consumption profiles between HPs and nHPs, analyse the relationship between FS use and socioeconomic/professional characteristics and assess the influence of nutritional knowledge and healthy lifestyle (exercise, balanced diet) on FS consumption. We aim to answer the following research question: What are the main factors influencing FS consumption in Portuguese adults, and how do they differ between HPs and nHPs? Ultimately, this study seeks to profile FS consumers in Portugal, understand their motivations, and determine if differences exist between HPs and nHPs, contributing to potential health interventions.

### 2.2. Study Design

**Figure 1:**
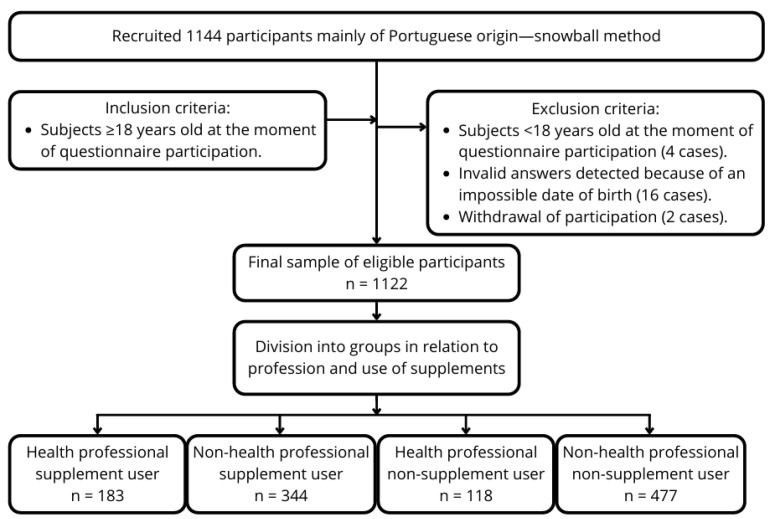
A flowchart showing the subjects of this study.

This cross-sectional study was conducted among the adult Portuguese population, who were invited to participate in answering a questionnaire. A flowchart showing the subjects of this study is provided in Figure 1. Data were collected between 30 June and 30 September 2023. The snowball sampling method, a non-probabilistic sampling method, was used to enrol the study participants, whereby participants helped spread the word, recruiting additional respondents. The method was described previously [22]. First, this study was published on the author’s personal and professional social media channels (i.e., LinkedIn^®^, Facebook^®^, and Instagram^®^), and requests were sent by discussion forums, professional organisations, mailing lists of students and teachers, and human resources departments of several national companies. The importance of sharing was reinforced in all requests on participants’ personal and professional social networks and also in professional and personal WhatsApp^®^ groups. This way, the chance of reaching a more diverse population increased. The investigators extended invitations to eligible participants who indicated their interest in participating in this study, which allowed them to fill out an online self-administered questionnaire.

Overall, a total of 1144 respondents participated voluntarily in this study. The inclusion criteria were (1) Portuguese, (2) aged 18 years and above, and (3) agreed to give informed consent. Those who met the inclusion criteria were eligible to participate in this study. Twenty-two participants were excluded. Twenty participants were excluded because they did not meet the age criteria: four because they were less than 18 years old at the moment of questionnaire participation and sixteen because they reported an invalid date of birth. Two participants declared no desire to participate.

After data collection, the study sample was divided according to occupation: (1) “healthcare professionals” (HPs) included nutritionists and Dietitians, pharmacists, medical doctors, Dentists, nurses, Veterinary Doctors, Physiotherapists, diagnostic and therapeutic technicians, and other professional therapies (e.g., acupuncture, osteopath, etc.) based on the question “What is your profession?” and (2) “non-healthcare professionals”.

Each of the selected groups was divided into food supplement users and non-users. Finally, the total sample was divided into four groups: (1) healthcare professional supplement users (HPS), (2) healthcare professional non-supplement users (HPnS), (3) non-health professional supplement users (nHPS), and (4) non-health professional non-supplement users (nHPnS).

The characteristics of the collected study sample HPs, HPS, and HPnS and nHPs, nHPS, and nHPnS are presented in Appendix A, Table A1 and Table A2.

Supplement users were qualified as the subjects who answered “yes” to whether they had consumed an FS in the last 12 months when answering the questionnaire.

The Faculty of Medicine of the University of Coimbra Ethics Commission approved this study in June 2023 (process number CE-066/2023).

### 2.3. Data Collection Tools

An online self-administered questionnaire (Google^®^ Forms) was developed from the validated KomPAN^®^ dietary habits and nutrition beliefs questionnaire and relevant published questionnaires [23,24,25,26]. The KomPAN^®^ questionnaire consists of four sections: (1) dietary habits, (2) frequency of food consumption, (3) views on food and nutrition, and (4) lifestyle and personal information [27]. All frequencies of consumption that were collected in the questionnaire were evaluated in 6 categories (from “never” (1) to “a few times a day” (6)). For each food item, the categories of the frequency of consumption were converted to values reflecting the daily frequency consumption (the range 0–2 times/day) [25].

The adapted questionnaire was piloted and pretested among 67 individuals. It included sociodemographic information, dietary habits, and participants’ knowledge, awareness, and attitudes related to health, particularly FSs. Questions from the developed survey were translated into Portuguese. All answers considered for this study were based on closed responses in order to reduce confounding factors and mistakes during interpretation. The questionnaire took an average of 25 min to complete.

#### 2.3.1. Sociodemographic Information

The questionnaire included sociodemographic characteristics such as gender, date of birth, weight and height, nationality, education level, place of residence, people living in a house (specific children), perception of financial situation and quality of life, and profession.

Based on the collected data, socioeconomic condition (SES), as a significant determinant of many behaviours, was calculated based on the literature [28,29]. The SES index was calculated as the product of the numerical values assigned to the individual categories of each SES factor. The more favourable categories of SES factors were assigned higher numerical values. After that, the SES was logarithmically transformed, and the tertile SESI distribution was used to identify respondents with low, average, or high SES. The SES evaluating method is detailed as follows:

SES = log(A × B × C × D × E)

where A—place of residence; B—self-declared financial situation; C—self-declared quality of life; D—educational qualifications; and E—educational level.

A—Place of residence was coded on a 5-point scale: (1) other, (2) city between 20,000 and 100,000 inhabitants, (3) city with less than 20,000 inhabitants, (4) village, and (5) city with more than 100,000 inhabitants.

B—Self-declared financial situation was coded on a 3-point scale: (3) high class, (2) middle class, and (1) low class.

C—Self-declared quality of life was coded on a 5-point scale: (1) we live modestly—we do not have enough money for basic needs; (2) we live modestly—we are very careful with our daily expenses; (3) we live normally—we have enough money for daily expenses, but we have to plan superfluous expenses; (4) we live relatively comfortably—we have enough money for our daily needs without a specific budget; and (5) we live very comfortably—we can enjoy some luxuries daily.

D—Educational qualifications were coded on a 4-point scale: (1) primary school, (2) less than secondary educational, (3) more than secondary education, and (4) higher education (e.g., Master’s degree, Doctorate).

E—Educational level. The highest level of studies achieved was coded on a 7-point scale: (1) basic education, (2) other, (3) high school, (4) Master’s degree, (5) Postgraduate, (6) university education, and (7) Doctorate.

An example of calculation for the SWS is as follows: SES = log(1 × 2 × 3 × 2 × 6) = log(72) = 4277 (value < 1 tercile; low socioeconomic status).

#### 2.3.2. Dietary Habits Information

Diet quality indices were determined for the defined products and product groups—a prohealthy diet index (pHDI-20), a nonhealthy diet index (nHDI-26), and a diet quality index (DQI) [27]. The DQI considers the foods in the pHDI-20 and nHDI-26 groups. The indicators were modified regarding the number of product groups for this study, as was performed in former studies [30]. The results were calculated based on the previously recommended division and interpreted according to the KomPAN^®^ protocol [26,31].

$$Diet\;Index\;(nHDI,\;pHDI,\;DQI)=\frac{100\%\times \sum \;A}{\sum \;B}\;[\%]$$

where *A* is the total of the reported daily intake for all items within specified food groups. At the same time, *B* represents the sum of the maximum possible daily intake for those same foods, calculated for a single product as 2.

#### 2.3.3. Health and FS Information

Information was collected regarding the participants’ knowledge, awareness, and attitudes towards health, specifically towards FSs. Several questions were raised, of which we emphasised the presence of chronic disease and taking medication, physical activity, and smoking use. Specifics about FS were collected regarding use, reasons for taking, places where they were bought, and advice. Information was also collected regarding knowledge about the definition of FSs.

### 2.4. Data Analysis

After considering the confidence level (99%) and the margin of error (3.42%), the calculated minimum sample size was 624 subjects, with a pre-determined margin of error of 5% and a 27% response distribution for each question (because we estimated that we had 26,6% of FS users in Portugal, according to official data) [5]. The required sample size was calculated using a sample size calculator (www.calculator.net) at a minimum 99% confidence level and 5%. The minimum required sample size of this study was 525, considering the adult population in Portugal (10,883,512) in 2023 [32,33].

All the variables were checked for normality using the Kolmogorov–Smirnov test. The χ^2^ test was used to assess the distribution of the categorical variables. The study groups’ characteristics involved calculating percentages, frequencies, means, standard deviations, medians, and confidence intervals, which were set at 95%.

A logistic regression analysis was conducted to identify significant correlations between supplement use and selected lifestyle traits. The dependent variable was supplement use (user vs. non-user), while the independent variables included lifestyle factors that may influence or be associated with the decision to use FSs, such as household type, education level, and socioeconomic status. To minimise the confounding variables’ influences, the results were adjusted on age and BMI. A *p*-value ≤ 0.05 was considered statistically significant, and a *p*-value ≤ 0.001 was considered highly important.

The calculations were performed using Statistica v.14.1 (StatSoft Polska sp. z o.o., Kraków, Poland) software.

## 3. Results

Of the 1122 survey participants, 301 (27%) were healthcare professionals (HPs). The general characteristics of this study subpopulation are shown in Table A1. The HPs were Portuguese (96.7%), female (83.7%), mainly had a high education level (university and PhD) (98%), and employed (100%); this group mainly reported a middle-class financial situation (89.7%), a normally and relatively comfortable quality of life (78.8%), and a high or average socioeconomic condition (SES, 35.2% and 42.9%, respectively). At the time of this study, most of the sample were nonsmokers (91.7%), had an average BMI (Body Mass Index (weight/height^2^)) of 23.0 +/− 3.7 kg/m^2^, and were aged 39. The common place of residence (34.2%) was a city (>100,000 inhabitants). Most HPs (58.8%) did not have children living in their houses, and 33.9% had four housemates.

Of these 301 healthcare professionals, 183 (60.8%) were FS users (HPS), and 118 (39.2%) were non-food supplement users (HPnS). These two groups were also characterised, as shown in Table A1. The statistics show that there was a significant difference between using FSs and nFSs in smoking. In the HP group (*p* = 0.04), we observed a significantly more frequent declaration of smoking. Significance related to the number of housemates and consumption of FSs (*p* = 0.017) was also observed; subjects used more FSs in smaller homes.

Of the study sample, 821 (73%) were non-healthcare professionals (nHPs). The general characteristics of this study subpopulation are shown in Table A2. In this group, as well, most of the respondents were Portuguese (96.6%), female (76.7%), had a high school or university education level (including PhD) (92.7%), were students (47.4%), and reported a middle-class financial situation (87.2%) and a normal and relatively comfortable quality of life (77.1%).

The SES status obtained subdivided the sample into three parts, representing low (36.1%), average (32.8%), and high (31.2%). Most of this sub-sample’s age was 18–33 (59.3%). The most common place of residence (36.3%) was a village. Most (64.3%) did not have children living in their houses, and 65.2% had three or four housemates. Of these 821 non-healthcare professionals, 344 (41.9%) were supplement users (nHPS), and 477 (58.1%) were non-supplement users (nHPnS). The statistical analysis showed a significant relation between nHP age and being an FS user (*p* = 0.036), revealing that non-supplement users were significantly younger (*p* > 0.01). FS use was also related to employment status; nHPnS mainly comprised full-time workers (*p* = 0.004). The statistics show a significant relationship between using FSs and socioeconomic status (SES, *p* = 0.035).

The probability of using FSs in Portuguese health professionals (HPs) and non-health professionals (nHPs) and their relations to the examined traits were adjusted for age and BMI. The results are presented in Table 1 and Table 2.

The analysis shows that the HP group living in small households had almost double the likelihood of being supplement users. Having a very good nutritional knowledge increased the probability of using FSs by more than 2 times; a nutritional knowledge level lower than very good reduced the likelihood of using FSs by 54%. Also, the declared profession of “pharmacist” seemed to increase the chance of using supplements by almost three times. Completely different results were observed among respondents whose declared profession was “medical doctor”. In this group, being a medical doctor reduced the likelihood of using an FS by 52%. Our analysis also revealed that medical doctor recommendations reduced the likelihood of using supplements in the HP group by 72%. A similar analysis was performed with the non-health professional (nHP) group.

The adherence to being an FS user was checked regarding education level, household size, nutritional knowledge, smoking, sports engagement, diet quality, SES, the person who provided a recommendation, and the medicines’ uses. As shown in Table 2, education at the primary and secondary levels decreased the adherence to belong to a group taking FSs by as much as 33%. Similarly, like in the HP group, living in a small household increased the likelihood of using FSs by 42%. We recognise that insufficient nutritional knowledge decreased the adherence to belong to a group using FSs by 51%. Conversely, having very good nutrition knowledge increased the probability of using an FS by 2.4 times. The results show that smoking in the past reduced the likelihood of using an FS by 41%. Engaging in sports was a significant factor in nHPs, which increased the likelihood of using supplements by 62%. A low value of diet quality index decreased the adherence to belong to a group taking FSs by 37%. High-intensity consumption of prohealthy foods reduced the chance of using supplements. A medical doctor’s recommendation reduced the likelihood of using FSs by 47%. Interestingly, being on maternity leave, unemployed, and a housewife/man at home increased the probability of using FS by almost 7 times.

There was no statistical significance between taking medicines and being an FS user. We also noted that proper knowledge of FSs did not positively influence FS intake in either the HP or nHP group.

## 4. Discussion

This study investigated the relationship between FS use and key driving factors among Portuguese individuals. Our findings reveal several essential points: (1) FSs are very popular in this group, where 47% of the responders to our questionnaire had consumed an FS in the last 12 months; (2) distinct factors influence FS use in health professionals (HPs) compared to non-health professionals (nHPs); (3) socioeconomic and professional characteristics are associated with FS consumption; and (4) eating habits and nutrition knowledge influence FS use, where higher knowledge correlates with increased consumption.

Our 47% FS usage rate is notably higher than the 29.2% reported in the National Portuguese Survey [5], suggesting a potential underestimation in the national data. Our study revealed a higher predominance of food supplement (FS) use in both health professionals (HPs, 60.8%) and non-health professionals (nHPs, 41.9%) compared to several other studies. While previous research in Portugal has characterised FS users, it often lacks detailed information on socioeconomic characteristics, professional profiles, and eating habits. Recent Portuguese studies concentrate on specific populations, broadly categorised as athletes and non-athletes. Within the athletic group, studies have examined FS use among soccer players, esports athletes, and gym attendees [20,34,35,36,37]. Among non-athletes, research has focused on groups like vegans [38] and medical doctors [13,39]. For instance, approximately one-third (32.3%) of esports players (Portuguese and Brazilian individuals living in Portugal) reported FS consumption [36], and less than half (43.8%) of gym users reported using at least one FS in the previous year [35]. A systematic review by Portuguese researchers highlighted significant FS use among female athletes [34]. Although comparable data from similar populations are limited, a recent Spanish study found a much higher prevalence (84.1%) of FS use in a comparable group [37]. Portuguese subpopulations exhibited higher FS use than the 29.2% reported in the Portuguese National Survey [5]. The higher prevalence in our sample (both HPs and nHPs exceeded the rates in these specialised studies) could be attributed to the higher education level within our groups (98.0% HPs and 59.4% nHPs). Furthermore, the discrepancy between our findings and the National Survey may stem from variations in the definition and identification of FSs, potentially leading to medication confusion. A clear and consistent definition of FS is crucial for accurate consumption assessments.

We observed striking differences in the factors driving FS use between HPs and nHPs. Several of the factors studied are exclusive to the HP group and increase HPs’ adherence to belong to a group taking an FS. Good knowledge about nutrition and being a pharmacist compared to other professions were positively associated with FS use, possibly reflecting pharmacists’ greater exposure to FS information through training and interactions with suppliers. Conversely, being a medical doctor, receiving a doctor’s recommendation, and having lower nutrition knowledge were negatively associated with FS use in this group. This may indicate a greater reliance on traditional medical practices and a potential distrust of FSs among doctors due to limited formal training on these products. There are no other statistically significant results in the HP group. In nHPs, high nutrition knowledge and a reported high consumption of healthy foods and low consumption of unhealthy foods were strong predictors of FS use. It is worth noticing that a doctor’s recommendation significantly decreased the likelihood of FS use in both HPs (72%) and nHPs (48%), contradicting the common perception that healthcare professionals are primary sources of FS recommendations: nutritionists (42.2%), medical doctors (23.5%), esports athletes [36], and nutritionists (23.1%) in gym members [35]. This unexpected finding warrants further investigation into the context of these recommendations and the potential influence of other factors.

Socioeconomic characteristics also played a role. Individuals living in smaller houses were likelier to use FSs in both groups. While socioeconomic status (SES) alone did not significantly predict FS use, professional characteristics did. HPs were more likely to consume FSs than nHPs; pharmacists were particularly likely, while doctors were less likely. This reinforces that health literacy and access to FS information, particularly within the pharmacy setting, may contribute to FS use.

Finally, our results confirm the link between healthy lifestyle habits and FS use. Higher scores on diet quality indices (a high diet quality index, a low nonhealthy diet index, and a high prohealthy diet index) were associated with an increased adherence to belonging to a group taking FSs in nHPs. Playing sports also increased the likelihood of consuming FSs. So, in the nHP group, healthy lifestyle habits seem to impact the decision to consume an FS.

Additionally, we can see that food consumption influences the choice to use an FS. The relationship between a healthy lifestyle and higher FS consumption is consistent with the available evidence. In nHPU, there is a statistically significant relationship between the use of FSs and the reported practice of physical activity. Previous studies with athlete populations reveal the high prevalence of FS use in these populations [20,34,35,36,37]. Also, better-assessed eating habits, the determination of which was previously described in this section, lead to greater consumption. Those results were aligned with research demonstrating the correlation between increased FS consumption and better dietary choices or adherence to the Mediterranean Diet [36,37,40].

Limited data exist on FS use obtained in national nutrition surveys, highlighting a gap in national nutrition monitoring. Data on FS consumption are crucial for characterising usage patterns and examining diet–health relationships [15,41]. In Europe, food supplements (FSs) are defined as foods with concentrated sources of nutrients or other substances with a nutritional or physiological effect, marketed in small, measured doses, such as tablets and liquids [42,43,44]. Their composition can include various nutrients and ingredients like micronutrients, amino acids, fibre, plants, and herbal extracts. FSs are intended to correct nutritional deficiencies, maintain adequate nutrient intake, or support specific physiological functions [42,43,44]. Critically, they are not medicines and, therefore, cannot perform pharmacological, immunological, or metabolic actions, nor are they intended to treat or prevent human diseases or modify physiological function in that therapeutic sense [42,43,44]. Despite their widespread availability, the use of FSs is not without potential risks due to often insufficient product oversight. Therefore, studying FS consumption in the general population and understanding the key factors driving their use is essential [45]. This knowledge will inform the development of evidence-based recommendations for healthcare and sports professionals to promote safe and effective FS use, particularly among vulnerable populations such as athletes, vegetarians, the elderly, and individuals taking multiple medications (polymedicated).

### 4.1. Perspective for Clinical Practice

This study’s findings have important implications for HPs, particularly within the evolving field of clinical practice. Lifestyle clinical practice uses evidence-based lifestyle intervention—including nutrition, physical activity, sleep, optimisation, and social support—to prevent chronic diseases. The increasing use of FSs has several important implications for clinical practice, and health professionals play a pivotal role in guiding patients’ FS choices and understanding the benefits and risks associated with FSs. Our results underscore the need for HPs to comprehensively understand FSs and their role in supporting overall health and nutrition, especially in the context of chronic disease management. Our findings also reveal a significant association between professional roles and FS use. Specifically, being a pharmacist nearly tripled the likelihood of consuming FSs, while being a medical doctor reduced this likelihood by half. These results can be interpreted in several ways. We hypothesise that higher health literacy increases health concerns, consequently leading to increased FS consumption. In Portugal, community pharmacists receive extensive information about FSs through training provided by suppliers. This direct exposure and training may contribute to their increased FS use. Conversely, medical doctors who are primarily focused on prescribing medications often receive limited information or training about FSs. This lack of formal education on FSs could lead to distrust and consequently reduce their consumption. This divergence in FS use between pharmacists and doctors highlights the need for targeted educational interventions. Providing evidence-based knowledge on FS empowers HPs to make well-informed decisions, which is an important strategy for ensuring the safe and effective use of these foods by patients and the general population [46]. In addition, research data indicate many patients utilise FSs without consulting a healthcare professional for therapeutic reasons. Integrating FS information into patient care helps mitigate interaction risks, optimise therapeutic and nutritional outcomes, and promote human health. Given the high prevalence of FS use in Portugal, HPs should be prepared to (i) provide evidence-based guidance for HPs to stay up to date on the latest studies on FSs, (ii) address patient misconceptions about FSs, viewing them as quick fixes or substitutes for healthy lifestyle practices, (iii) integrate FSs into personalised care plans, which can be a valuable tool for patients with specific nutritional deficiencies or those seeking to support certain physiological functions, and (iv) monitor the potential risks of FSs through HP surveillance, namely, by screening patients for potential adverse effects or interactions associated with the use of FSs, especially in polymedicated populations, such as the elderly and people with chronic diseases. Medical doctors, pharmacists, nutritionists, nurses, and other HPs each bring unique expertise to patient care. A collaborative approach allows for a more holistic assessment of the patient’s needs, including their dietary habits, medication use, and overall health status. With their in-depth knowledge of medications and potential drug–nutrient interactions, pharmacists can play a critical role in identifying potential risks associated with FS use. Nutritionists can provide expert guidance on dietary modifications and ensure FSs are integrated appropriately within a balanced diet. Doctors can assess the patient’s overall health status and determine if FS use is appropriate, given any existing medical conditions. Surgical doctors and nurses should be trained to avoid complications and side effects due to FSs in the surgical period; discontinuation periods and drug interactions are necessary to prevent and solve problems that may occur due to FSs used by the patient [47]. This interprofessional approach in different health areas is essential for ensuring patient safety and promoting informed decision-making regarding FS use [46]. This approach will safeguard patient health, enhance the quality of healthcare, and reduce health costs. A crucial consideration for the advancement of FS research lies in the imperative to ensure inclusivity and representativeness across diverse demographics, extending beyond a focus solely on individuals experiencing illness [45].

### 4.2. Limits

The present study, with a substantial sample size of 1122 participants and minimal exclusion criteria, acknowledges the potential for selection bias. Its cross-sectional design precludes causal inferences. Furthermore, the online snowball sampling methodology may limit the generalisability of the findings to the broader Portuguese population. To mitigate this limitation, this study provides a comprehensive sociodemographic characterisation of the sample, dividing it into healthcare professional (HP) and non-healthcare professional (nHP) subpopulations for more detailed analysis. This approach allows comparisons with existing Portuguese studies, highlighting concordant results and offering critical analyses of divergent findings.

A further limitation stems from the self-reported nature of dietary data, introducing the possibility of reporting and recall bias. This study also notes the disproportionate representation of women (79%) compared to the 2023 Portuguese adult female population (53%) [48], a common issue in online surveys. However, within the HP subpopulation, the female representation (84%) more closely aligns with the 2023 data for female healthcare professionals in the Portuguese national health system (78%) [49], suggesting a more representative gender balance within this specific group. This nuanced analysis of gender representation strengthens this study’s findings within the HP subpopulation.

How the questionnaire was answered may also create some bias in the results, as the older age group has some limitations in accessing information technology, resulting in a lower response rate among older people.

While acknowledging these limitations, this study’s strengths, including its large sample size and detailed subpopulation analysis, contribute valuable insights to the existing literature.

## 5. Conclusions

This study investigates the key factors influencing FS use in Portuguese adults, addressing the research question of what factors drive FS consumption and how they differ between healthcare professionals (HPs) and non-healthcare professionals (nHPs). Contrary to the expectation that FS use would be higher among individuals with poorer dietary habits, the results revealed a positive association between healthy lifestyle choices and FS consumption. Participants with better eating habits (higher consumption of nutritious foods, lower consumption of unhealthy foods) and those with more physical activity were more likely to use FSs. This trend was observed across both the HP and nHP groups, suggesting a potential misinterpretation of the purpose of FSs, which are intended to complement, not replace, a healthy diet.

The lack of statistical significance between the HP recommendation for individual FS consumption raises concerns about public awareness of potential risks associated with FS use, particularly drug interactions. Within the HP group, pharmacists exhibited higher FS consumption than medical doctors, potentially indicating that increased training, knowledge, and access to information about FSs lead to more informed and rational use. This highlights the influence of professional characteristics on FS consumption.

All these findings significantly impact the understanding of FS use in Portugal and inform future research and public health initiatives. Specifically, they underscore the need for further investigation into the complex relationship between dietary patterns and FS use, including motivations for use and perceived benefits. Future research should also explore the potential influence of specific nutritional deficiencies on FS choices. Furthermore, this study reinforces the importance of robust training for healthcare professionals on the appropriate use, benefits, and potential risks of FSs, enabling them to provide accurate and practical guidance to the public.

## Figures and Tables

**Table 1:** Odds ratios (ORs with 95% confidence intervals (95% CIs)) of intake supplements to the selected traits (education and nutritional knowledge level; sociodemographic traits; diet quality; the recommendation source) in Portuguese health professionals (HPs).

	n (%)	^1^ HPS—Without Adjustment	^1^ HPS—Adjusted for BMI	^1^ HPS—Adjusted for BMI and Age
		OR (CI 95%); *p*	OR (CI 95%); *p*	OR (CI 95%); *p*
Primary and secondary education	295 (98%)	0.64 (0.13; 3.24); *p* = 0.59	0.68 (0.13; 3.51); *p* = 0.65	0.68 (0.13; 3.53); *p* = 0.64
Living in small household (one and two people)	107 (36%)	1.99 (1.20; 3.31); *p* < 0.01	1.97 (1.19; 3.28); *p* < 0.01	1.97 (1.19; 3.28); *p* < 0.01
Living in bigger household (three people and more)	194 (65%)	0.50 (0.30; 0.83); *p* < 0.01	0.51 (0.31; 0.84); *p* < 0.01	–
Insufficient nutritional knowledge level	10 (3%)	0.64 (0.18; 2.25); *p* = 0.48	0.62 (0.17; 2.21); *p* = 0.46	0.62 (0.17; 2.21); *p* = 0.46
Nutrition knowledge at insufficient or sufficient level	109 (36%)	0.73 (0.45; 1.18); *p* = 0.20	0.73 (0.45; 1.18); *p* = 0.19	0.73 (0.45; 1.18); *p* = 0.19
Nutritional knowledge level lower than very good	245 (81%)	0.45 (0.23; 0.87); *p* < 0.05	0.46 (0.24; 0.89); *p* < 0.05	0.46 (0.24; 0.89); *p* < 0.05
Nutritional knowledge at a very good level	56 (19%)	2.21 (1.15; 4.28); *p* < 0.05	2.18 (1.13; 4.23); *p* < 0.05	2.19 (1.13; 4.23); *p* < 0.05
Smoking in the past	76 (25%)	0.55 (0.24; 1.28); *p* = 0.16	0.52 (0.19; 1.42); *p* = 0.20	0.51 (0.17; 1.52); *p* = 0.23
Engaging in sports	177 (59%)	1.44 (0.90; 2.31); *p* = 0.13	1.43 (0.90; 2.30); *p* = 0.13	1.43 (0.89; 2.30); *p* = 0.13
Low value of diet quality index (^2^ DQI)	58 (19%)	1.07 (0.59; 1.93); *p* = 0.83	1.07 (0.59; 1.94); *p* = 0.81	1.08 (0.59; 1.95); *p* = 0.81
High value of diet quality index (^2^ DQI)	137 (46%)	1.38 (0.86; 2.21); p = 0.18	1.37 (0.86; 2.20); *p* = 0.18	1.37 (0.86; 2.20); *p* = 0.18
Low value of nonhealthy diet index (^3^ nHDI)	115 (38%)	0.84 (0.52; 1.36); p = 0.48	0.83 (0.51; 1.34); *p* = 0.44	0.83 (0.51; 1.34); *p* = 0.44
High value of nonhealthy diet index (^3^ nHDI)	87 (29%)	1.16 (0.69; 1.94); *p* = 0.58	1.15 (0.69; 1.93); *p* = 0.59	1.15 (0.69; 1.93); *p* = 0.59
Low value of prohealthy diet index (^4^ pHDI)	68 (23%)	0.66 (0.38; 1.14); *p* = 0.13	0.67 (0.39; 1.16); *p* = 0.15	0.67 (0.39; 1.16); *p* = 0.15
High value of prohealthy diet index (^4^ pHDI)	122 (41%)	1.33 (0.82; 2.14); *p* = 0.25	1.31 (0.81; 2.11); *p* = 0.27	1.31 (0.81; 2.11); *p* = 0.27
Low SES value (first quartile)	56 (19%)	0.69 (0.39; 1.25); *p* = 0.22	0.70 (0.39; 1.26); *p* = 0.23	0.69 (0.38; 1.26); *p* = 0.23
High SES value (fourth quartile)	84 (28%)	0.93 (0.55; 1.56); *p* = 0.78	0.92 (0.55; 1.55); *p* = 0.76	0.92 (0.54; 1.56); *p* = 0.75
Doctor’s recommendation	47 (16%)	0.30 (0.14; 0.66); *p* < 0.01	0.30 (0.13; 0.66); *p* < 0.01	0.28 (0.12; 0.62); *p* < 0.01
Being a pharmacist	150 (50%)	2.61 (1.59; 4.27); *p* < 0.001	2.62 (1.62; 4.25); *p* < 0.001	2.81 (1.70; 4.63); *p* < 0.001
Being a doctor	47 (16%)	0.51 (0.27; 0.95), *p* < 0.05	0.49 (0.26; 0.92), *p* < 0.05	0.48 (0.25; 0.91), *p* < 0.05
Knowledge of food supplements	65 (22%)	0.59 (0.34; 1.03); *p* = 0.06	0.59 (0.34; 1.04); *p* = 0.07	0.59 (0.34; 1.04); *p* = 0.065
Taking medicine	85 (28%)	0.89 (0.53; 1.49; *p* = 0.66	0.92 (0.55; 1.56); *p* = 0.76	0.92 (0.54; 1.57); *p* = 0.75

^1^ HPS—healthcare professional supplement users; ^2^ DQI—diet quality index; ^3^ nHDI—nonhealthy diet index; ^4^ pHDI—prohealthy diet index.

**Table 2:** Odds ratios (ORs with 95% confidence intervals (95% CIs)) of intake supplements to the selected traits (education and nutritional knowledge level; sociodemographic traits; diet quality; the recommendation source) in Portuguese non-health professionals (nHPs).

Factor	n (%)	^1^ nHPS—Without Adjustment	^1^ nHPS—Adjusted for BMI	^1^ nHPS—Adjusted for BMI and Age
		OR (CI 95%); *p*	OR (CI 95%); *p*	OR (CI 95%); *p*
Primary and secondary education	333 (41%)	0.79 (0.59; 1.05); *p* = 0.10	0.77 (0.58; 1.02); *p* = 0.07	0.67 (0.49; 0.91); *p* < 0.01
Living in small household (one and two people)	189 (23%)	1.25 (0.90; 1.73); *p* = 0.19	1.27 (0.91; 1.76); *p* = 0.16	1.42 (1.00; 2.01); *p* < 0.05
Living in bigger household (three people and more)	632 (77%)	0.80 (0.58; 1.12); *p* = 0.19	0.79 (0.57; 1.10); *p* = 0.16	0.71 (0.50; 1.00); *p* < 0.05
Insufficient nutritional knowledge level	87 (11%)	0.49 (0.30; 0.81); *p* < 0.01	0.49 (0.30; 0.80); *p* < 0.01	0.49 (0.30; 0.81); *p* < 0.01
Nutrition knowledge at insufficient or sufficient level	528 (64%)	0.51 (0.38; 0.68); *p* < 0.001	0.51 (0.38; 0.69); *p* < 0.001	0.52 (0.39; 0.69); *p* < 0.001
Nutritional knowledge level lower than very good	778 (95%)	0.41 (0.22; 0.77); *p* < 0.01	0.41 (0.22; 0.77); *p* < 0.01	0.42 (0.22; 0.78); *p* < 0.01
Nutrition knowledge at a very good level	43 (5%)	2.45 (1.30; 4.63); *p* < 0.01	2.46 (1.30; 4.65); *p* < 0.01	2.41 (1.28; 4.56); *p* < 0.01
Smoking in the past	309 (38%)	0.68 (0.49; 0.93); *p* < 0.05	0.65 (0.46; 0.91); *p* < 0.05	0.59 (0.40; 0.87); *p* < 0.01
Engaging in sports	402 (49%)	1.57 (1.19; 2.08); *p* < 0.01	1.58 (1.19; 2.09); *p* < 0.01	1.62 (1.22; 2.15); *p* < 0.001
Low value of diet quality index (^2^ DQI)	314 (38%)	0.64 (0.48; 0.86); *p* < 0.01	0.65 (0.49; 0.87); *p* < 0.01	0.63 (0.47; 0.85); *p* < 0.01
High value of diet quality index (^2^ DQI)	241 (29%)	1.37 (1.01; 1.85); *p* < 0.05	1.36 (1.00; 1.84); *p* < 0.05	1.37 (1.01; 1.86); *p* < 0.05
Low value of nonhealthy diet index (^3^ nHDI)	250 (31%)	1.33 (0.99; 1.80); *p* = 0.06	1.36 (1.00; 1.83); *p* < 0.05	1.49 (1.09; 2.04); *p* < 0.05
High value of nonhealthy diet index (^3^ nHDI)	284 (35%)	0.88 (0.65; 1.17); *p* = 0.37	0.86 (0.64; 1.16); *p* = 0.32	0.81 (0.60; 1.10); *p* = 0.17
Low value of prohealthy diet index (^4^ pHDI)	303 (37%)	0.82 (0.62; 1.10); *p* = 0.19	0.84 (0.63; 1.12); *p* = 0.23	0.83 (0.62; 1.12); *p* = 0.22
High value of prohealthy diet index (^4^ pHDI)	250 (31%)	1.43 (1.06; 1.93); *p* < 0.05	1.41 (1.05; 1.91); *p* < 0.05	1.41 (1.05; 1.91); *p* < 0.05
Low SES value	250 (31%)	0.96 (0.71; 1.30); *p* = 0.79	0.97 (0.72; 1.32); *p* = 0.85	0.94 (0.69; 1.28); *p* = 0.70
High SES value	164 (20%)	0.92 (0.65; 1.30); *p* = 0.63	0.92 (0.65; 1.30); *p* = 0.62	0.99 (0.69; 1.42); *p* = 0.94
Doctor’s recommendation	165 (20%)	0.55 (0.35; 0.84); *p* < 0.01	0.53 (0.35; 0.83); *p* < 0.01	0.53 (0.35; 0.83); *p* < 0.01
Knowledge of food supplements	216 26%	0.70 (0.51; 0.97); *p* < 0.05	0.72 (0.52; 1.00); *p* < 0.05	0.74 (0.53; 1.03); *p* = 0.07
Taking medicine	209 (26%)	1.07 (0.78; 1.46; *p* = 0.69	1.11 (0.80; 1.53); *p* = 0.53	1.21 (0.86; 1.69); *p* = 0.28
Employment—on maternity leave; I am unemployed or other (housewife/man at home)	17 (2%)	6.70 (1.91; 23.55); *p* < 0.01	6.92 (1.96; 24.38); *p* < 0.01	6.86 (1.95; 24.18); *p* < 0.01

^1^ nHPS—non-healthcare professional supplement users; ^2^ DQI—diet quality index; ^3^ nHDI—nonhealthy diet index; ^4^ pHDI—prohealthy diet index.

## Data Availability

The data presented in this study are available on request from the corresponding author. The data are not publicly available due to privacy restrictions.

## Supplementary Materials

The following supporting information can be downloaded at https://www.mdpi.com/article/10.3390/foods14050884/s1, Table S1. Supplementary data for variables (data source and assessment methods).

## Appendix A

The characteristics of the collected study sample HPs, HPS, and HPnS and nHPs, nHPS, and nHPnS are presented in Appendix A Table A1, Table A2 and Table S1.

**Table A1:** Characteristics of the study sample health professionals (HPs).

Variable	HPs ^1^	HPS ^2^	HPnS ^3^	Chi ^2^
	n = 301	n = 183 (60.8%)	n = 118 (39.2%)	
	Mean ± SD (min–max)	Mean ± SD (min–max)	Mean ± SD (min–max)	
BMI (kg/m^2^)	23.0 ± 3.7 (16.9–41.0)	22.9 ± 3.7 (16.9–41.0)	23.2 ± 3.6 (17.6–34.0)	-
Age (years)	39 ± 11 (23–71)	38 ± 10.8 23–61	39 ± 11.3 (23–71)	-
Age categorisation (years)	n (%)	n (%)	n (%)	
18–22	0 (0.0)	0 (0.0)	0 (0.0)	
23–33	117 (38.9)	70 (38.3)	47 (39.8)	NS 3.44
34–44	86 (28.6)	54 (29.5)	32 (27.1)	
45–55	76 (25.2)	46 (25.1)	30 (25.4)	
56–66	20 (6.6)	13 (7.1)	7 (5.9)	
>66	2 (0.7)	0 (0.0)	2 (1.7)	
	Mean ± SD (min–max)	Mean ± SD (min–max)	Mean ± SD (min–max)	
Status SES (-)	5.45 ± 0.89 (2.08–7.50)	5.45 ± 0.85 (2.77 ± 7.50)	5.44 ± 0.94 (2.08 ± 7.09)	NS
	n (%)	n (%)	n (%)	
Status SES (categorisation)				
Low	66 (21.9)	37 (20.2)	29 (24.6)	NS 3.27
Average	129 (42.9)	86 (47.0)	43 (36.4)	
High	106 (35.2)	60 (32.8)	46 (39.0)	
Gender				
Male	49 (16.3)	31 (16.3)	18 (15.3)	NS 0.15
Female	252 (83.7)	152 (83.7)	100 (84.7)	
BMI (kg/m^2^)				
Underweight (<18.5)	11 (3.7)	9 (4.9)	2 (1.7)	NS 3.74
Normal (18.5–24.99)	217 (72.1)	130 (71.0)	87 (73.7)	
Overweight (≥25–30)	56 (18.6)	36 (19.7)	20 (16.9)	
Obese (≥30)	17 (5.6)	8 (4.4)	9 (7.6)	
Education level				
Primary school	4 (1.3)	2 (1.1)	2 (1.7)	
High school	2 (0.7)	1 (0.5)	1 (0.8)	
University	277 (92.0)	168 (91.8)	109 (92.4)	NS 0.56
PhD	18 (6.0)	12 (6.6)	6 (5.1)	
Self-declared financial situation				
Low class	18 (6.0)	11 (6.0)	7 (5.9)	NS 0.41
Middle class	270 (89.7)	163 (89.1)	107 (90.7)	
High class	13 (4.3)	9 (4.9)	4 (3.4)	
Self-declared quality of life				
Modestly poor	1 (0.3)	1 (0.5)	0 (0.0)	NS 3.10
Modestly	32 (10.6)	20 (10.9)	12 (10.2)	
Normally	135 (44.9)	79 (43.2)	56 (47.5)	
Relatively comfortably	102 (33.9)	67 (36.6)	35 (29.7)	
Comfortably	31 (10.3)	16 (8.7)	15 (12.7)	
Employment				
Full-time worker	287 (95.3)	174 (95.1)	113 (95.8)	NS 0.08
Temporary job	14 (4.7)	9 (4.9)	5 (4.2)	
Smoking cigarettes				
Yes	25 (8.3)	20 (10.9)	5 (4.2)	(0.04) 4.21
No	276 (91.7)	163 (89.1)	113 (95.8)	
Nationality				
Portugal	291 (96.7)	177 (96.7)	114 (96.6)	NS 0.0027
Others	10 (3.3)	6 (3.3)	4 (3.4)	
Place of residence				
Village inhabitants	68 (22.6)	38 (20.8)	30 (25.4)	NS 3.91
City (<20,000) inhabitants	44 (14.6)	28 (15.3)	16 (13.6)	
City (20,000–100,000) inhabitants	86 (28.6)	59 (32.2)	27 (22.9)	
City (>100,000) inhabitants	103 (34.2)	58 (31.7)	45 (38.1)	
Number of housemates				
1	36 (12.0)	28 (15.3)	8 (6.8)	(0.017) 11.95
2	71 (23.6)	48 (26.2)	23 (19.5)	
3	62 (20.6)	29 (15.8)	33 (28.0)	
4	102 (33.9)	58 (31.7)	44 (37.3)	
≥5	30 (10.0)	20 (10.9)	10 (8.5)	
Children living in a house				
0	177 (58.8)	111 (60.7)	66 (55.9)	NS 2.87
1–2	108 (35.9)	60 (32.8)	48 (40.7)	
≥3	16 (5.3)	12 (6.6)	4 (3.4)	

^1^HPs—total healthcare professionals; ^2^ HPS—healthcare professional supplement users; ^3^ HPnS—healthcare professional non-supplement users.

**Table A2:** Characteristics of the study sample non-health professionals (nHPs).

Variable	nHPs ^1^	nHPS ^2^	nHPnS ^3^	Chi ^2^
	n = 821	n = 344 (41.9%)	n = 477(58.1%)	
	Mean ± SD (min–max)	Mean ± SD (min–max)	Mean ± SD (min–max)	
BMI (kg/m^2^)	23.3 ± 3.68 (14.1–40.1)	23.1 ± 3.65 (14.1–40.1)	23.5 ± 3.7 (17.4–37.0)	-
Age (years)	33.3 ± 14.53 (18.0–85.0)	32.1 ± 13.66 (18.0–75.0)	34.2 ± 15.09 (18.0–85.0)	-
Age categorisation (years)	n (%)	n (%)	n (%)	
18–22	313 (38.1)	129 (37.5)	184 (38.6)	(0.036) 11.90
23–33	174 (21.2)	90 (26.2)	84 (17.6)	
34–44	112 (13.6)	41 (11.9)	71 (14.9)	
45–55	147 (17.9)	59 (17.2)	88 (18.4)	
56–66	57 (6.9)	21 (6.1)	36 (7.5)	
>66	18 (2.2)	4 (1.2)	14 (2.9)	
	Mean ± SD (min–max)	Mean ± SD (min–max)	Mean ± SD (min–max)	
Status SES (-)	5.17 ± 1.06 (1.10–7.65)	5.23 ± 0.96 (2.08 ± 7.03)	5.13 ± 1.13 (1.10 ± 7.65)	
Status SES	n (%)	n (%)	n (%)	
Low	296 (36.1)	108 (31.4)	188 (39.4)	(0.035) 6.73
Average	269 (32.8)	127 (36.9)	142 (29.8)	
High	256 (31.2)	109 (31.7)	147 (30.8)	
Gender				
Male	187 (22.8)	84 (24.4)	103 (21.6)	NS 3.16
Female	630 (76.7)	257 (74.7)	373 (78.2)	
Non-binary	3 (0.4)	2 (0.6)	1 (0.2)	
I do not want to report	1 (0.1)	1 (0.3)	0 (0.0	
BMI (kg/m^2^)				
Underweight (<18.5)	44 (5.4)	21 (6.1)	23 (4.8)	NS 3.15
Normal (18.5–24.9)	555 (67.6)	237 (68.9)	318 (66.7)	
Overweight (≥25.0–30.0)	171 (20.8)	70 (20.3)	101 (21.2)	
Obese (≥30.0)	51 (6.2)	16 (4.7)	35 (7.3)	
Education level				
Primary school	60 (7.3)	21 (6.1)	39 (8.2)	NS 4.49
High school	273 (33.3)	107 (31.1)	166 (34.8)	
University	456 (55.5)	205 (59.6)	251 (52.6)	
PhD	32 (3.9)	11 (3.2)	21 (4.4)	
Self-declared financial situation				
Low class	91 (11.1)	42 (12.2)	49 (10.3)	NS 3.84
Middle class	716 (87.2)	293 (85.2)	423 (88.7)	
High class	14 (1.7)	9 (2.6)	5 (1.0)	
Self-declared quality of life				
Modestly poor	9 (1.1)	3 (0.9)	6 (1.3)	NS 1.30
Modestly	115 (14.0)	44 (12.8)	71 (14.9)	
Normally	382 (46.5)	163 (47.4)	219 (45.9)	
Relatively comfortably	251 (30.6)	105 (30.5)	146 (30.6)	
Comfortably	64 (7.8)	29 (8.4)	35 (7.3)	
Employment				
Full-time worker	349 (42.5)	134 (39.0)	215 (45.1)	(0.004) 15.43
Temporary job	40 (4.9)	16 (4.7)	24 (5.0)	
On maternity leave, unemployed, or other	17 (2.1)	14 (4.1)	3 (0.6)	
Student	389 (47.4)	172 (50.0)	217 (45.5)	
Retired or receiving disability benefit	26 (3.2)	8 (2.3)	18 (3.8)	
Smoking cigarettes				
Yes	144 (17.5)	63 (18.3)	81 (17.0)	NS 0.25
No	677 (82.5)	281 (81.7)	396 (83.0)	
Nationality				
Portugal	793 (96.6)	334 (97.1)	459 (96.2)	NS 0.46
Others	28 (3.4)	10 (2.9)	18 (3.8)	
Place of residence				
Village inhabitants	298 (36.3)	123 (35.8)	175 (36.7)	NS 6.23
City (<20,000) inhabitants	84 (10.2)	42 (12.2)	42 (8.8)	
City (20,000–100,000) inhabitants	216 (26.3)	98 (28.5)	118 (24.7)	
City (>100,000) inhabitants	223 (27.2)	81 (23.5)	142 (29.8)	
Number of housemates				
1	49 (6.0)	21 (6.1)	28 (5.9)	NS 3.72
2	140 (17.1)	66 (19.2)	74 (15.5)	
3	274 (33.4)	117 (34.0)	157 (32.9)	
4	261 (31.8)	106 (30.8)	155 (32.5)	
≥5	97 (11.8)	34 (9.9)	63 (13.2)	
Children living in a house				
0	528 (64.3)	235 (68.3)	291 (61.0)	NS 4.40
1–2	274 (33.4)	103 (29.9)	171 (35.8)	
≥3	19 (2.3)	6 (1.7)	13 (2.7)	
High	247 (30.1)	107 (31.1)	140 (29.4)	

nHPs ^1^—total non-healthcare professionals. nHPS ^2^—non-healthcare professional supplement users; nHPnS ^3^—non-healthcare professional not supplement users.
