# Supplementary material for: Key Factors Driving Portuguese Individuals to Use Food Supplements—Findings from a Cross-Sectional Study
Source: Foods. 2025 Mar 5;14(5):884. doi: 10.3390/foods14050884 (PMC11899158; doi:10.3390/foods14050884)
Supplement: Supplementary file 1 [file foods-14-00884-s001.zip › foods-3475864-supplementary.pdf]

Table S1. Supplementary data for variables (data source and assessment methods)

Variables of interest	Data Sources	Assessment Methods
**The Main question for subpopulation divisions:**		
Healthcare Professional (HP) Status	Self-reported information through survey question: **"What's your profession?"**	multiple choice question
Food Supplement User	Self-reported information through survey question: **"Have you taken any food supplements in the last 12 months?"**	multiple choice question
**Questions for Characteristics of the Study Sample**		
Age	Self-reported information through survey "Fill date of birth": day, month, year.	informatic equation converting date of birth to age using the date of questionnaire answer.
Status Socioeconomic Status (SES)		SES construction is detailed in the main document
Gender:	Self-reported information through survey question: "What is your gender?"	multiple choice question
Body Mass Index (BMI)	Likely calculated from self-reported height and weight.	Calculated using the standard BMI formula (weight in kg/height in m^2).
Education Level	Self-reported information through survey question: "Indicate your educational qualifications, if you are currently studying, indicate which cycle you are attending: "	multiple choice question
Financial Situation	Self-reported information through survey question: "How do you describe your financial situation?"	multiple choice question
Quality of Life	Self-reported information through survey question: "How do you describe the quality of life in your home?"	multiple choice question
Employment Status	Are you working?	multiple choice question
Smoking Cigarettes	Self-reported information through survey question: "Have you smoked cigarettes, pipes or other types of tobacco in the past?"	multiple choice question

Nationality	Self-reported information through survey question: "What is your nationality?"	(Write the name of your country of birth e.g. Portugal)?"
Place of Residence	Self-reported information through survey question: "What is your place of residence?"	multiple choice question
Number of Housemates	Self-reported information through survey question: "How many people live in your house (including yourself)?"	multiple choice question
Children Living at Home	Self-reported information through survey question: How many children/teenagers live in your house? (People under 18 years old)	multiple choice question
**Additional questions for Odds ratios of intake FS to the select traits.**		
Sports Engagement	Self-reported information through survey question: "Do you practice any sports?"	multiple choice question
Diet Quality Index (DQI)		DQI construction is detailed in the main document.
Nutritional Knowledge	Self-reported information through survey question: "How do you describe your knowledge about nutrition"	multiple choice question
Medical Doctor Recommendation	Self-reported information through survey question: "Who recommended the food supplements to you?"	multiple choice question
Medicines Use	Self-reported information through survey question: "Do you take any medication chronically?"	multiple choice question
Knowledge about FS	Self-reported information through survey question: What are food supplements for you?"	multiple choice question
